# Prognosis of COVID-19 pneumonia can be early predicted combining Age-adjusted Charlson Comorbidity Index, CRB score and baseline oxygen saturation

**DOI:** 10.1038/s41598-022-06199-3

**Published:** 2022-02-11

**Authors:** Pilar Nuevo-Ortega, Carmen Reina-Artacho, Francisco Dominguez-Moreno, Victor Manuel Becerra-Muñoz, Luis Ruiz-Del-Fresno, Maria Antonia Estecha-Foncea, HOPE Group Hospital Universitario Virgen de la Victoria, A. M. Aguilar-Galvez, R. Barrera-Serrano, Victor Manuel Becerra-Muñoz, E. Cabrera-Cesar, J. M. Castillo-Caballero, S. Cordon-Alvarez, F. Cota-Delgado, D. Daga-Ruiz, A. De La Torre-Muñoz, Francisco Dominguez-Moreno, V. Doncel-Abad, Maria Antonia Estecha-Foncea, E. Estevez-Escobar, A. Fernandez-Villalba, S. Garcia-Aragon, M. C. Garcia-Cruz, I. G. Garcia-Gomez, A. M. Gomez-Perez, P. Gonzalez-Redondo, P. Lara-Dominguez, P. Martinez-Lopez, A. Martinez-Mesa, M. Mateos-Rodriguez, G. Moratalla-Cecilia, B. Murcia-Casas, M. Nieto-Gonzalez, Pilar Nuevo-Ortega, C. Perez-Lopez, A. Puerto-Morlan, Carmen Reina-Artacho, J. Rodriguez-Capitan, C. Rueda-Molina, Luis Ruiz-Del-Fresno, C. Salazar-Ramirez, L. Salido-Diaz, E. Sanchez-Alvarez, A. Sanchez-Calderon, A. Sanchez-Garcia, F. Segura-Gonzalez, M. Valera-Rubio, A. Vallejo-Baez, M. C. Vera-Sanchez, N. A. Zamboschi

**Affiliations:** 1 Intensive Care Unit, Hospital Universitario Virgen de la Victoria, Málaga, Spain; 2 Instituto de Investigación Biomédica de Málaga, Málaga, Spain; 3 Instituto de Salud Carlos III, Madrid, Spain; 4 Internal Medicine, Hospital Universitario Virgen de la Victoria, Málaga, Spain; 5 Pneumology Hospital Universitario Virgen de la Victoria, Málaga, Spain; 6 Endocrinology, Hospital Universitario Virgen de la Victoria, Málaga, Spain; 7 Cardiology, Hospital Universitario Virgen de la Victoria, Málaga, Spain; 8 Hospital Pharmacy, Hospital Universitario Virgen de la Victoria, Málaga, Spain; 9 Infectious Disease Unit, Hospital Universitario Virgen de la Victoria, Málaga, Spain; 10 Anesthesiology, Hospital Universitario Virgen de la Victoria, Málaga, Spain

**Keywords:** Public health, Viral infection, Respiratory tract diseases

## Abstract

In potentially severe diseases in general and COVID-19 in particular, it is vital to early identify those patients who are going to progress to severe disease. A recent living systematic review dedicated to predictive models in COVID-19, critically appraises 145 models, 8 of them focused on prediction of severe disease and 23 on mortality. Unfortunately, in all 145 models, they found a risk of bias significant enough to finally "not recommend any for clinical use". Authors suggest concentrating on avoiding biases in sampling and prioritising the study of already identified predictive factors, rather than the identification of new ones that are often dependent on the database. Our objective is to develop a model to predict which patients with COVID-19 pneumonia are at high risk of developing severe illness or dying, using basic and validated clinical tools. We studied a prospective cohort of consecutive patients admitted in a teaching hospital during the “first wave” of the COVID-19 pandemic. Follow-up to discharge from hospital. Multiple logistic regression selecting variables according to clinical and statistical criteria. 404 consecutive patients were evaluated, 392 (97%) completed follow-up. Mean age was 61 years; 59% were men. The median burden of comorbidity was 2 points in the Age-adjusted Charlson Comorbidity Index, CRB was abnormal in 18% of patients and basal oxygen saturation on admission lower than 90% in 18%. A model composed of Age-adjusted Charlson Comorbidity Index, CRB score and basal oxygen saturation can predict unfavorable evolution or death with an area under the ROC curve of 0.85 (95% CI 0.80–0.89), and 0.90 (95% CI 0.86 to 0.94), respectively. Prognosis of COVID-19 pneumonia can be predicted without laboratory tests using two classic clinical tools and a pocket pulse oximeter.

## Introduction

In potentially severe diseases in general, and COVID-19 in particular, it is vital to early identify those patients who are going to progress to severe disease. Physicians often care for patients who present a clearly mild or severe profile and do not need to calculate a predictive index to make decisions. However, in other cases, it is not easy to anticipate the clinical course, and that is when a prediction rule can be helpful, e.g., in a patient with mild to moderate acute symptoms, but with a weak baseline situation; or in a patient who does have striking acute symptoms but is young and healthy. It can also be helpful for healthcare managers when need to quantify the healthcare demand that it is going to be faced and prepare the necessary resources in advance. Finally, a suitable predictive rule would be useful as a quality control tool for both, clinical physicians, and healthcare managers^1^.

Motivated by the urgent need to characterise COVID-19 there has been a blast of publications (more than 80,000 up to early December 2020); the symptoms and initial characteristics of the disease are well known, but the determinants of its course are less clear. A living systematic review dedicated to predictive models in COVID-19^1^, in its latest version (search updated May 5), has found 145 models, 8 of them focused on prediction of severe disease and 23 on mortality. Unfortunately, in all 145 models, they found a risk of bias significant enough to finally "not recommend any for clinical use". The most frequent bias issues referred to the analysis; however, the most serious ones were those related to sampling. Authors recommend concentrating on avoiding biases in sampling and prioritising the study of already identified predictive factors, rather than the identification of new ones that are often dependent on the database. Our objective is to develop a model to predict which patients with COVID-19 pneumonia are at high risk of developing severe illness, using readily available clinical data in the absence of laboratory or sophisticated computing/artificial intelligence.

## Methods

### Study design

Prospective cohort study, formed by all patients consecutively admitted to the Hospital Universitario Virgen de la Victoria (HUVV) with COVID-19 pneumonia, during the first wave: March 1 to April 28, 2020. Follow-up lasted until the discharge of the last patient: July 21, 2020. HUVV is a 506-bed hospital, classified as level 2, located in Málaga (southern Spain), which directly serves a population of 470,000 inhabitants.

### Participants and source of data

*The inclusion criteria* were: confirmed, symptomatic SARS-CoV-2 infection; and requiring hospital admission. *Exclusion criteria* were: age under 14 years. When the patient had consulted several times at the Emergency Department, data was collected from the consultation in which the acute infection by SARS-CoV-2 was diagnosed.

*SARS-CoV-2 infection was confirmed* by real-time reverse transcription polymerase chain reaction (RT-PCR), or detection of IgM antibodies with enzyme immunoassay techniques (ELISA).

*Hospital admission* was based on respiratory symptoms plus radiological infiltrates or significant comorbidity. Radiologists examined the plain chest radiographs of patients suspected of having SARS-CoV-2 infection. Admission to Intensive Care Unit (ICU) was based on the development of severe disease and recoverability.

Data were collected from patients or their relatives, the computerised medical record, and the daily handover list of unstable COVID patients in the wards. It was collected within the framework of "International COVID-19 Clinical Evaluation Registry: HOPE-COVID 19" that was evaluated by the Ethics and Research Committee of the Hospital Clínico San Carlos in Madrid. The database records were entered anonymized, with an alphanumeric code and the identifying data were kept in a different file guarded by the local researchers; following data protection laws in force: Ley Orgánica 15/1999, of December 13, de Protección de Datos de Carácter Personal; Ley 41/2002, of November 14, Básica Reguladora de la Autonomía del Paciente y Derechos y Obligaciones en materia de Información y Documentación Clínica. Ley 14/2007, of July 3, de Investigación Biomédica; and Ethical Principles for Medical Research on Human Beings established in the Declaration of Helsinki by the World Medical Association. Written informed consent was waived by the Ethics and Research Committee of the Hospital Clínico San Carlos, due to the nature of the anonymized registry and the severity of the situation.

Model development and reporting followed the TRIPOD (Transparent Reporting of a multivariable prediction model for Individual Prediction Or Diagnosis) guidelines^2^.

### Patients and public involvement

Patients and public have not been involved in the development of the research question, outcome measures, design nor execution of this study.

### Outcomes

*The primary outcome* was the development of severe disease, defined by the presence of one of the following criteria: a respiratory failure that needs an inspiratory fraction of oxygen (FiO_2_) equal to or greater than 0.6, shock or severe dysfunction of another organ, or death. *The secondary outcome* was vital status at hospital discharge (alive/dead).

### Predictors

In each patient, we collected demographic characteristics (gender, age, provenance), comorbidities, baseline functional situation and usual medication, the situation at admission (symptoms and signs, complementary examinations), evolution during hospitalisation, and status at discharge.

As an indicator of acute physiological injury, we calculated the CRB scale^3,4^ on admission (Supplementary Material); it is a validated version of the CURB-65 scale^5^; endorsed by British Thoracic Society^6^ and NICE^7,8^. Arterial hypotension and tachypnea were defined as in CRB score: systolic arterial pressure < 90 mmHg or diastolic arterial pressure ≤ 60 mmHg; tachypnea was defined by a respiratory rate ≥ 39 per minute. Fever was defined as temperature ≥ 38 °C. At admission, radiologists reported the chest radiographs of patients with suspected SARS-CoV-2 infection.

As a summary variable of comorbidity, we calculated the Age-Adjusted Charlson Comorbidity Index^9,10^ (Age-Charlson) (Supplementary Material). We chose to evaluate age as a part of the comorbidity index instead of in CRB-65 for two reasons: first, because of clinical significance, as we consider that increasing age provides information about non-explicit comorbidity, it is a kind of "hidden comorbidity index"; and second, for statistical reasons, to minimise the number of predictor variables while maximising the exploitation of a continuous variable.

### Statistical analysis

The sample size was determined by the evolution of the pandemic. No imputation of values has been made in the missing data.

In the descriptive analysis, absolute and relative frequencies were calculated in the categorical variables, mean and standard deviation (SD) in the continuous ones with Normal distribution, and median and interquartile range (IQR) in the continuous ones with non-Normal distribution.

For the bivariate analysis, according to the outcomes of interest, we calculated p values with Chi-squared, Student’s t or Fisher’s exact tests as appropriate. P value less than 0.05 are considered statistically significant, no adjustment for multiple comparations were done. All test were 2-tailed.

Multivariate analysis was carried out with forward conditional stepwise logistic regression. Dependent variables were the primary or secondary outcomes; independent variables were selected by clinical and statistical criteria in several stages. For statistical analysis, we used the IBM SPSS Statistics package, version 25.

### Ethics approval and consent to participate

This study has been done within the framework of "International COVID-19 Clinical Evaluation Registry: HOPE-COVID 19" project that was approved by the Ethics and Research Committee of Hospital Clínico San Carlos in Madrid(20/241-E) and Agencia Española de Medicamentos y productos Sanitarios (EPA- = D). The database records were entered anonymized, with an alphanumeric code and the identifying data were kept in a different file guarded by the local researchers; following data protection laws in force: Ley Orgánica 15/1999, of December 13, de Protección de Datos de Carácter Personal; Ley 41/2002, of November 14, Básica Reguladora de la Autonomía del Paciente y Derechos y Obligaciones en materia de Información y Documentación Clínica. Ley 14/2007, of July 3, de Investigación Biomédica; and Ethical Principles for Medical Research on Human Beings established in the Declaration of Helsinki by the World Medical Association. Written informed consent was waived because the characteristics of the anonymized registry and the severity of the situation.

## Results

**Figure 1:**
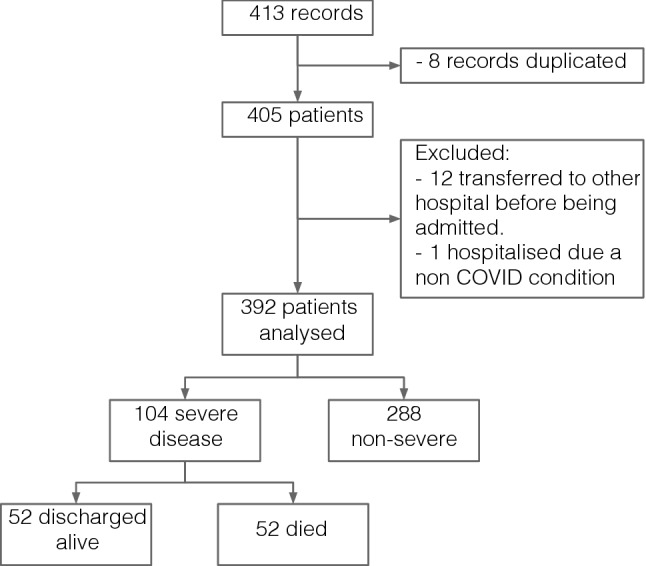
Participants flowchart.

The first COVID-19 patient was admitted to our hospital on March 1, 2020, and the last of this "first wave" on April 28, 2020; during that period 413 records were included in the database. From them, eight records were deleted because of duplication; twelve patients were excluded because they were transferred to another hospital due to administrative reasons, without being admitted at HUVV; and one patient was excluded because he had an asymptomatic SARS-CoV-2 infection and was hospitalised because of non-related condition (atrioventricular block). Therefore, 392 patients with COVID-19 are analysed. Figure 1 shows the participants flowchart. Follow-up lasted until the discharge of the last patient: July 21, 2020. One hundred and four patients developed severe disease (27% of the study group), and fifty-two died (13%). In the Supplementary Material, Fig. S1 shows the daily flow of admissions, discharges and patients hospitalised.

Baseline characteristics are shown in Table 1. The mean age was 61 years; 59% were men. The median burden of comorbidity was 2 points in the Age-Charlson scale, being significantly higher in patients who developed severe disease (median 4.5 versus 2 in the non-severe), and in those who died (median 6.5 points versus 2 in those who survived). Fourteen per cent of the patients had some degree of dependency in activities of daily living. The most prevalent pathological background was arterial hypertension (46%). In the bivariate analysis, the variables most clearly associated with the development of severe disease or death were: age (71 years in the severe vs 57 in the non-severe), cerebrovascular disease, chronic heart disease and arterial hypertension.

**Table 1:** Baseline characteristics and home medication, according to the development of severe disease and vital status at discharge.

Variables	Disease severity			Vital status at discharge		
	Moderate	Severe	*p*-value	Alive	Dead	*p*-value
	n: 288	n: 104		n: 340	n: 52	
**Demographics**						
Gender (masculine)	159 (55)	71 (68)	***0.020***	196 (58)	34 (65)	0.291
Age (mean ± SD) (n: 392)	57 ± 16	71 ± 12	** < *0.001***	59 ± 15	75 ± 11	** < *0.001***
**Comorbidities**						
Chronic lung disease (n: 233)	40 (28)	32 (36)	0.222	48 (26)	24 (52)	** < *0.001***
Chronic heart disease (n: 371)	23 (9)	33 (32)	** < *0.001***	35 (11)	21 (40)	** < *0.001***
Arterial Hypertension (n: 392)	107 (37)	72 (69)	** < *0.001***	138 (41)	41 (79)	** < *0.001***
Obesity (n: 204)	18 (13)	19 (30)	***0.003***	24 (14)	13 (41)	** < *0.001***
Diabetes mellitus (n: 375)	34 (12)	34 (36)	** < *0.001***	51 (16)	17 (34)	***0.002***
Chronic kidney disease (n: 392)	10 (3)	13 (13)	***0.001***	12 (4)	11 (21)	** < *0.001***
Cerebrovascular disease (n: 389)	15 (36)	27 (64)	** < *0.001***	25 (7)	17 (33)	** < *0.001***
Connective tissue disease (n: 383)	6 (2)	2 (2)	0.969	6 (2)	2 (4)	0.325
Chronic liver disease (n: 385)	8 (3)	7 (7)	0.075	10 (3)	5 (10)	***0.019***
Cancer (n: 389)	23 (8)	12 (12)	0.290	27 (8)	8 (15)	0.083
Immunodepression (n: 379)	13 (5)	7 (7)	0.306	13 (4)	7 (14)	***0.002***
Age-Charlson score, median (IQR) (n: 392)	2 (0–3.5)	4.5 (3–6.5)	** < *0.001***	2 (1–4)	6.5 (4.7–7.5)	** < *0.001***
Dependency activities of daily living (n: 383)	26 (9)	27 (28)	** < *0.001***	30 (9)	23 (46)	** < *0.001***
**Home medications**						
Antiplatelets (n: 380)	38 (13)	31 (33)	** < *0.001***	48 (14)	21 (44)	** < *0.001***
Anticoagulants (n: 382)	11 (4)	16 (16)	** < *0.001***	16 (5)	11 (22)	** < *0.001***
ARA-II or ACE inhibitors (n: 386)	85 (30)	58 (59)	** < *0.001***	109 (33)	34 (67)	** < *0.001***
Beta blockers (n: 385)	25 (9)	24 (24)	0,570	33 (10)	16 (31)	0,096
Vitamin D supplements (n: 381)	14 (5)	6 (6)	** < *0.001***	15 (5)	5 (10)	** < *0.001***

ARA: angiotensin II receptor antagonists. ACEi: angiotensin-converting enzyme inhibitor. In each variable, total number of valid data is specified in the first column. In each outcome, absolute frequency, percentage by outcome and p-values for the comparation of moderate vs severe disease and alive vs dead at discharge are shown.

In bold and italic those associations that are statistically significant.

IQR: interquartile range; SD: standard deviation.

The situation on arrival at the Emergency Department is summarised in Tables 2 and 3. The average duration of symptoms was 7 days (median), being significantly shorter in patients with clinical deterioration (6 days in those who developed severe disease, and 5 days in those who eventually died). The CRB score was 0 points in more than 80% of the cases, but any increase was strongly associated with adverse evolution. Baseline pulse oximetry saturation on arrival was the simple complementary examination most strongly associated with a negative outcome in the unadjusted bivariate analysis.

**Table 2:** Symptoms and signs at presentation.

Variables	Disease severity				Vital status at discharge		
	Moderate		Severe	p-value	Alive	Dead	p-value
	n: 288		n: 104		n: 340	n: 52	
Days from symptoms onset to Hospital admission, median (IQR)	7 (4–10)		6 (3–8.2)	***0.002***	7 (4–10)	5 (3–7.2)	***0.013***
**Symptoms and signs**							
Asymptomatic (n: 385)	12 (92%)		1 (8%)	0.135	13 (100%)	0 (0%)	0.152
Fever (n: 381)	231 (76%)		73 (24%)	0.290	265 (87%)	39 (13%)	0.735
Cough (n: 375)	216 (77%)		66 (23%)	0.196	252 (89%)	30 (11%)	***0.029***
Dyspnea (n: 380)	129 (66%)		66 (34%)	** < *0.001***	161 (83%)	34 (17%)	***0.018***
Tachypnea (n: 375)	16 (33%)		33 (67%)	** < *0.001***	32 (65%)	17 (35%)	** < *0.001***
Asthenia (n: 338)	132 (71%)		54 (29%)	0.212	159 (85%)	27 (15%)	0.591
Myalgias (n: 322)	126 (78%)		36 (22%)	0.273	143 (88%)	19 (12%)	0.832
Odynophagia (n: 274)	31 (94%)		2 (6%)	***0.008***	32 (97%)	1 (3%)	0.109
Diarrhoea (n: 337)	58 (79%)		15 (21%)	0.199	68 (93%)	5 (7%)	0.075
Anosmia (n: 211)	13 (100%)		0 (0%)	***0.027***	13 (100%)	0 (0%)	0.116
Dysgeusia (n: 208)	12 (92%)		1 (8%)	0.099	13 (100%)	0 (0%)	0.112
Thromboembolism (n: 379)	2 (14%)		12 (86%)	** < *0.001***	10 (71%)	4 (29%)	0.091
Haemoptysis (n: 380)	0 (0%)		4 (100%)	** < *0.001***	1 (25%)	3 (75%)	** < *0.001***
Heart failure (n: 379)	3 (13%)		20 (87%)	** < *0.001***	6 (26%)	17 (74%)	** < *0.001***
Baseline SpO_2_ < 90 (n: 392)	19 (28%)		50 (72%)	** < *0.001***	38 (55%)	31 (45%)	** < *0.001***
Glasgow coma score < 15 (n: 364)	5 (26%)		14 (74%)	** < *0.001***	7 (37%)	12 (63%)	** < *0.001***
Arterial hypotension (n: 362)	4 (33%)		8 (67%)	** < *0.001***	7 (58%)	5 (42%)	** < *0.001***
CRB score^a^ (n: 392)	0	264 (81%)	60 (19%)	** < *0.001***	298 (92%)	26 (8%)	** < *0.001***
	1	23 (40%)	35 (60%)		38 (66%)	20 (34%)	
	2	1 (13%)	7 (88%)		4 (50%)	4 (50%)	
	3	0 (0%)	2 (100%)		0 (0%)	2 (100%)	

According to the development of severe disease and vital status at discharge.

IQR: interquartile range. SpO_2_: arterial saturation by pulse oximetry. In each outcome, absolute frequency, percentage by outcome and p-values for the comparation of moderate vs severe disease and alive vs dead at discharge are shown.

^a^In the CRB score the vast majority of the patients had a score of zero (83%), which makes the quartiles and range very uninformative, so for a better description in the bivariate analysis we show the absolute and relative frequencies of all values (0–3); its p-value compares 0 points versus more than 0. In each variable, total number of valid data is specified in the first column. In bold and italic those associations that are statistically significant.

**Table 3:** Laboratory and radiology at admission. Bivariate analysis according to whether they developed severe disease, and status at discharge.

Variables	Disease severity			Vital status at discharge		
	Moderate	Severe	p-value	Alive	Dead	p-value
	n: 288	n: 104		n: 340	n: 52	
Haemoglobin (mmol/L) (n: 381)	8.7 ± 1.2	8.7 ± 1.2	0.194	8.63 ± 1.06	8.13 ± 1.3	***0.022***
Leukocytes (× 106/L) (n: 382)	6.6 ± 4.5	7.3 ± 4.1	0.178	6.7 ± 4.4	8 ± 5	0.225
Lymphocyte (× 106/L) (n: 380)	1.3 ± 0.6	1.0 ± 0.7	***0.003***	1.3 ± 0.7	0.9 ± 0.6	** < *0.001***
Platelets (× 106/L) (n: 381)	224 ± 90	202 ± 94	0.053	220 ± 90	200 ± 90	0.082
Sodium in plasma (mmol/L) (n: 378)	138 ± 5	138 ± 6	0.254	138 ± 5	138 ± 7	0.902
Creatinine > 0.11 mmol/L (n: 380)	37 (13%)	38 (39%)	** < *0.001***	48 (14%)	27 (57%)	** < *0.001***
LDH > 4.1 ukat/L (n: 371)	147 (53%)	77 (81%)	** < *0.001***	182 (57%)	42 (86%)	** < *0.001***
AST > 0.66 ukat/L (n: 339)	99 (39%)	41 (50%)	0.066	118 (39%)	22 (55%)	0.061
Troponin I > 0.05 mcg/L (n: 78)	3 (5%)	4 (20%)	***0.045***	5 (7%)	2 (25%)	0.094
CRP > 47.6 nmol/L (n: 382)	240 (84%)	95 (98%)	** < *0.001***	285 (86%)	50 (98%)	***0.016***
PCT > 500 ng/L (n: 264)	3 (2%)	13 (18%)	** < *0.001***	11 (5%)	5 (14%)	***0.034***
d-dimer > 0.5 mg/L (n: 331)	142 (58%)	65 (76%)	***0.002***	171 (59%)	36 (88%)	** < *0.001***
Ferritin > 0.72 nmol/L (n: 139)	42 (37%)	12 (48%)	0.300	49 (39%)	5 (42%)	0.834
Triglycerides > 1.69 mmol/L (n: 99)	12 (16%)	7 (30%)	0.118	16 (18%)	3 (30%)	0.360
Abnormal chest X-ray. (n: 379)	275 (97%)	93 (97%)	0.880	319 (97%)	49 (96%)	0.641

In each variable, total number of valid data is specified in the first column. In each outcome, absolute frequency and percentage by outcome; or mean and Standard deviation are shown; as well as p-values for the comparation of moderate vs severe disease and alive vs dead at discharge. In bold and italic those associations that are statistically significant.

**Figure 2:**
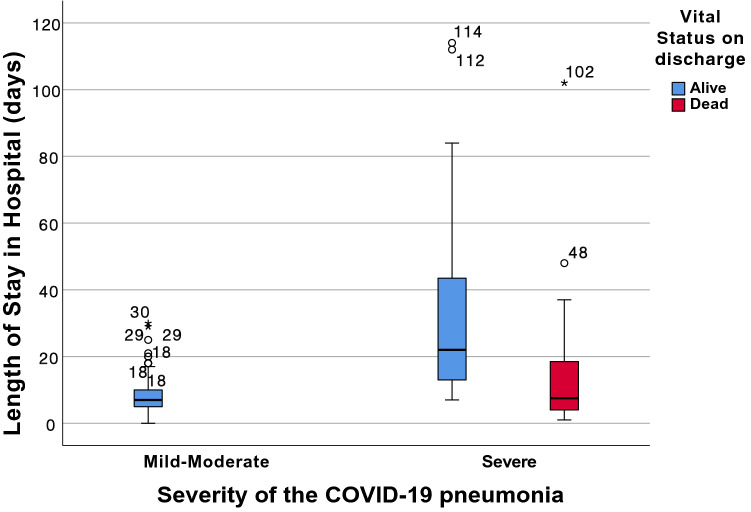
Relationship between length of stay in hospital, severity and vital status on discharge. Box plot showing the length of stay in hospital, according to the severity of the disease and status at discharge. Numbers in the graph area indicate length of stay.

During hospitalisation, most of the patients received hydroxychloroquine (92%) and lopinavir/ritonavir (80%). Drugs aimed at attenuating the inflammatory response (corticosteroids, tocilizumab, interferon) were used less frequently (around 20%) and preferentially in the most severely ill patients. Remdesivir was not administered due to availability issues (Table 4). One hundred and four patients developed severe disease (27% of the sample), at a median of 9 days from the onset of symptoms, forty (10% of the sample) were admitted to the ICU. Fifty-two (13%) died, sixteen of them in the ICU (40% of all admitted to ICU). The median hospital stay of the total sample was 8 days, with two clearly differentiated patterns: shorter stays in patients with moderate disease and in patients who die (median of 7 and 7.5 days respectively), and longer stays in patients who survived despite developing severe disease (median 22 days, IQR: 13–42.2); these differences are clinically, epidemiologically, and statistically significant (Fig. 2).

**Table 4:** Bivariate analysis of hospital treatment according to the development of severe disease and status at discharge.

Variables	Disease severity			Vital status at discharge		
	Moderate	Severe	p-value	Alive	Dead	p-value
	n: 288	n: 104		n: 340	n: 52	
Corticosteroids (n: 385)	34 (12%)	73 (70%)	< 0.001	75 (23%)	32 (62%)	< 0.001
Hydroxychloroquine (n: 387)	263 (93%)	93 (90%)	0.459	315 (94%)	41 (80%)	< 0.001
Lopinavir/ ritonavir (n: 387)	223 (79%)	85 (83%)	0.388	271 (81%)	37 (73%)	0.181
Interferon (n: 386)	24 (8%)	44 (43%)	< 0.001	54 (16%)	14 (27%)	0.048
Tocilizumab (n: 383)	0 (0%)	44 (43%)	< 0.001	28 (8%)	16 (31%)	< 0.001
High flow nasal prongs(n: 385)	0 (0%)	18 (18%)	< 0.001	9 (3%)	9 (18%)	< 0.001
Non-invasive ventilation (n: 387)	7 (2%)	13 (13%)	< 0.001	15 (4%)	5 (10%)	0.119
Invasive ventilation (n: 386)	0 (0%)	36 (35%)	< 0.001	20 (6%)	16 (31%)	< 0.001

In each variable, total number of valid data is specified in the first column. In each outcome, absolute frequency, percentage by outcome and p-values for the comparation of moderate vs severe disease and alive vs dead at discharge are shown. In bold and italic those associations that are statistically significant.

**Figure 3:**
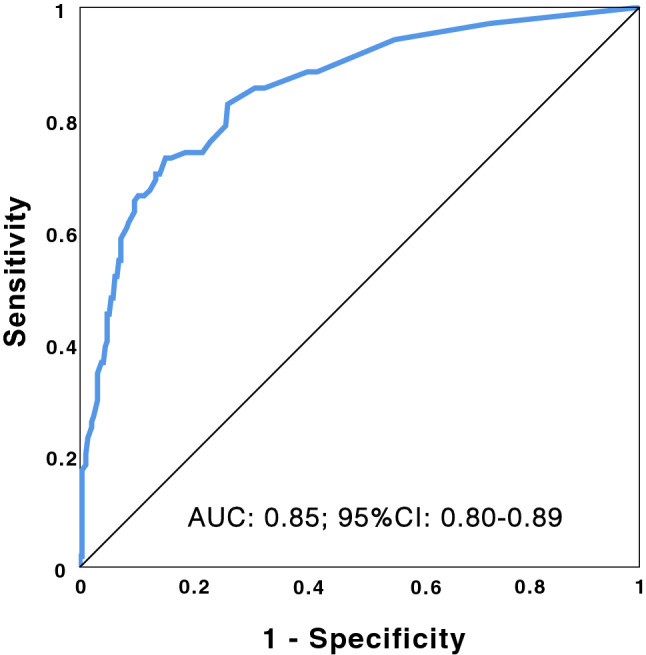
ROC curve of the severe disease prediction model.

*The final multivariate model for prediction of the primary outcome* (development of severe disease), is shown in Table 5. It contains only three variables: Age-Charlson scale, CRB scale, and baseline desaturation by pulse oximetry. There are no missing data, so 392 patients are analysed. Cox and Snell’s R^2^ is 0.28, and Nagelkerke’s 0.42; Hosmer–Lemeshow test p = 0.22; C statistic: 0.85 (95% CI 0.80–0.89), global sensitivity: 93%, specificity: 55%. Figure 3 displays the receiver operating characteristic curve (ROC curve). Logistic regression requirements are met. Among the rest of the factors that could have independent prognostic value, only CRP, LDH and heart failure had statistically significant coefficients but did not improve the overall performance of the model (Supplementary Material, Table S1 and Fig. S2). Gender, hypertension, previous dependence, days from onset of symptoms to arrival at the hospital, DD, AST, or acute renal failure upon admission were not significant; troponin, ferritin and PCT were not be evaluated in the multivariate analysis because there are few cases with valid data in the first day. We also do not evaluate inpatient drug therapy because their administration has been highly biased by the severity perceived by the physician assisting the patient, and it was not possible to control this confounding factor.

**Table 5:** Multivariate model for predicting the development of severe disease.

	B	Sig	OR	95%CI for OR	
				Lower	Upper
Age-Charlson	0.253	< 0.001	1.28	1.16	1.42
CRB	1.427	< 0.001	4.16	2.26	7.65
Baseline SpO_2_ < 90	1.866	< 0.001	6.46	3.32	12.53
Constant	− 2.676	< 0.001	0.069

**Figure 4:**
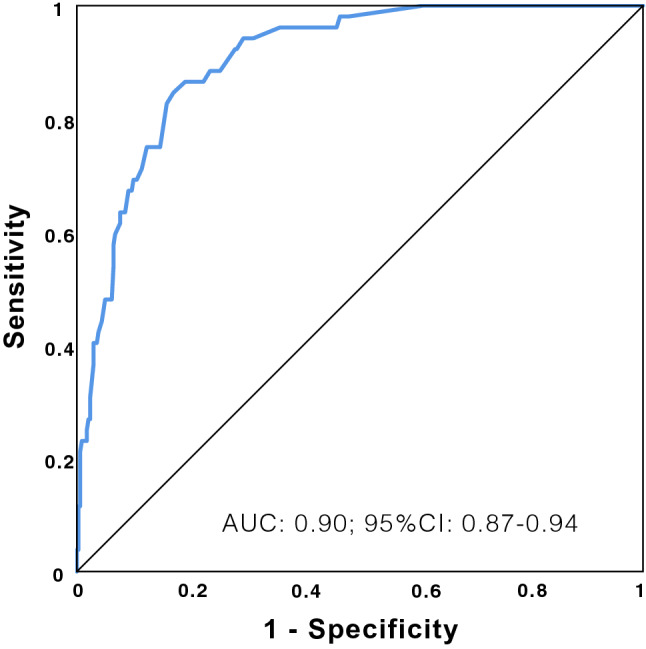
ROC curve of the predictive model of mortality.

*In the multivariate analysis for prediction of the secondary outcome* (death), we arrived at a model with the same predictors, and remarkably similar performance (Table 6, Fig. 4). Hosmer Lemeshow test p = 0.85; Cox and Snell’s R^2^: 0.24 and Nagelkerke’s 0.45. The C statistic: 0.90 (95% CI 0.86–0.94), overall sensitivity 97%, specificity 40%. Among the rest of the variables that could be independent risk factors, only LDH and lymphocyte count reached statistical significance but did not significantly improve the model (Supplementary Material, Table S2 and Fig. S3). Gender, arterial hypertension, dependence in activities of daily living, DD, CRP, PCT, AST, leukocytes, haemoglobin, platelets, sodium, acute renal failure or days from the onset of symptoms to arrival at the hospital were not significant; troponin or ferritin cannot be explored in the multivariate model because there are few cases with valid data.

**Table 6:** Multivariate model for predicting death.

	B	Sig	OR	95%CI for OR	
				Lower	Upper
Age-Charlson	0.424	< 0.001	1.52	1.32	1.76
CRB	1.091	0.001	2.97	1.55	5.69
Baseline SpO_2_ < 90	1.636	< 0.001	5.13	2.44	10.77
Constant	− 4.609	< 0.001	0.01

## Discussion

The main conclusion of this study is that the prognosis of a patient with COVID-19 pneumonia can probably be predicted by combining a widely validated comorbidity scale and an acute disease scale; the only "complementary examination" that we include is arterial saturation by pulse oximetry, a measurement that can be done at patient's home as easily as taking blood pressure. We have chosen the most popular comorbidity scale: the Charlson Comorbidity Index^11^ (age-adjusted version^9^); and as a pneumonia severity scale, one of the CURB-65 family: the CRB scale^3^; but surely there will be other options. The main point is to check the validity of an idea with such clinical coherence: the prognosis of a patient essentially depends on the balance between the resistance capacity and the aggressiveness of the acute problem.

*The sample* we study meets the requirements to be considered representative: confirmed cases, consecutively included, in the same phase of the disease (on admission to hospital), with homogeneous admission criteria, in a naturally delimited time frame, with prospective data collection and complete follow-up (minimal percentage of losses: 12/404, 3%). Furthermore, looking at the proportion of hospital beds occupied by COVID-19 patients (maximum 38%, Supplementary Material, Fig. S1), we get at the impression that the confounding effect that a possible work-overload could have on patient outcomes has been lower in our hospital than in other cases^12,13^.

*Baseline characteristics* also support the idea of representativeness; they are very similar to other series^12,14,15^, predominantly male, with a mean age of 60 years, similar to the USA^16^ and intermediate between that of China^17^ (around 55 years), and United Kingdom^18^ (70 years old). The comorbidity burden was low (Age-Charlson median 2 points), similar to that observed by Casas-Rojo^15^ in Spain, and in other series that have evaluated age and the Charlson index separately: Italy^19^, USA^20^, Denmark^21^, or China^13^. In all of them, with such different socio-geographic contexts, both characteristics were independent risk factors, which reinforces the idea of the suitability of combining them in Age-Charlson.

*In the first clinical evaluation,* CRB and SpO_2_ were abnormal in only 18% of the patients, but with a strong association with severity. SpO_2_ could be especially useful in COVID-19 patients, helping to detect what has been called "silent hypoxemia"^22,23^.

*The most widespread model in which data on comorbidity and acute disease are combined in patients with pneumonia* is the PSI scale^24^. However, it has a substantial disadvantage: it cannot be used outside a health centre since 7 of its 19 variables require laboratory or radiology/ultrasound. There are very few studies with predictive models applicable in primary care that, at the same time, implement such intuitive idea as that assessing the prognosis of potentially seriously ill patients requires considering not only the aggressiveness of the acute disease but also the burden of chronic disease that weakens them^25^. Generally, both components have been studied as alternatives^26^, and rarely as complementary^27,28^. In patients with COVID-19, Petrilli^29^ and ISARIC^18,30^ are two groups that more closely resemble this study’s objective. Petrilli does not explicitly include a comorbidity scale but empirically reaches the same conclusions: age, comorbidity, oxygenation and inflammation parameters determine the need for hospitalisation and the development of severe disease; the relative weight of each possibly varies depending on the outcome and the population of interest. ISARIC-4C is based on the components of the Charlson Index and CURB-65, along with gender, obesity and CRP to build a model with 8 predictor variables, including 2 biochemical which limits its application outside the hospital context; unexpectedly, hypotension has not reached the final model. In polypathological COVID-19 patients, the usefulness of combining acute damage and comorbidity scales has also been partially reported, in this case not with the Charlson index but with a specific scale for polypathological patients (PROFUND)^31^.

*Regarding other variables* that could be important, we have explored the baseline functional situation in terms of dependency for activities of daily living, and though it was significant in the unadjusted bivariate analysis (Table 1), it ceased to be so in the multivariate after incorporating the Age-Charlson scale; however, we think it deserves to be further explored. Casas-Rojo^15^ have similar results: 16% of dependency for activities of daily living, and association with worse evolution in the bivariate analysis; the multivariate analysis has yet to be published. Bernabeu-Wittel^31^ in a study focused on multiple pathological patients with COVID-19 incorporates functional status (Barthel index) into the assessment of comorbidity.

*The rate of severe disease* in this series is 27%; in other studies, it ranges between 15 and 37%^29,30,32,33^. This variability may be due to differences in the selected sample and in the definition of severe disease:

- Major differences in sampling: due to differences in the age of the patients (which we will address next); or due to exclusively including patients diagnosed by chest CT^34^ (which is more sensitive than plain radiography); or excluding patients who already present in a severe condition^35–38^ (because their objective is to study the progression from non-severe to severe); or limiting follow-up to a short period which does not allow that a significant proportion of included patients reach the outcome of interest^39,40^, and therefore rising a significant risk of selection bias that will be later discussed.
- Important differences in the definition of severe disease: most of predictive models developed in China^37,40,41^ use the definition recommended by the National Health Commission of China, that is broader than ours. In this Chinese definition, a ratio of arterial oxygen pressure to inspiratory oxygen fraction (Pa/Fi) less than 300 is a sufficient criterion to diagnose severe pneumonia. So, for example, a patient that with a FiO_2_ of 0.3 and a PaO_2_ of 80 mmHg (Pa/Fi: 80/0.3 = 267), should be considered severe with the Chinese definition, but not with ours. Our definition adopts criteria routinely recommended to consider the admission of a patient with pneumonia to an area of high dependency or an Intensive Care Unit^42–44^, regarding FiO_2_ it requires to need 0.6 or more. Other studies limit the definition to “admission in ICU or Intermediate Unit”^18^; overlooking that in order to admit a patient in these units, in addition to severity, patient recoverability and availability of beds are also assessed; this explains the variability in the use of ICUs and why a high percentage of severely ill patients are not treated in ICU^45^, approximately 60% in our series.

*Crude mortality rate* in our series is 13% (of hospitalised patients). Again, direct comparison with other series is difficult, even being mortality a more robust outcome than disease severity. In Spain, mortality in multicentre studies of hospitalised patients has been 21–28% ^15,32^, in the United Kingdom 30%^30^, Italy 20%^46^. Age distribution and incomplete follow-up are two factors that could explain not only differences in raw mortality but also in the performance of predictive models.

**Figure 5:**
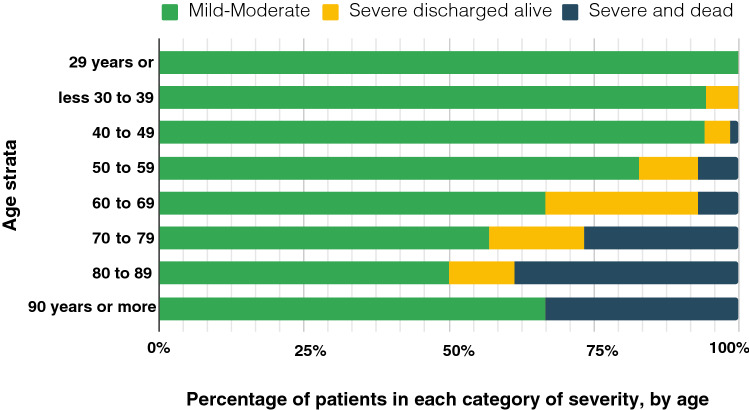
Percentage of severe disease and mortality, by age strata. Total patients in each decade: 29 years or less: 6 patients; 30–39: 35 patients; 40–49: 68 patients; 50–59: 70 patients; 60–69: 84 patients; 70–79: 79 patients; 80–89: 44 patients; 90 years or older: 6 patients.

*Mortality varies according to age in all series*; in our study, it ranges from 0% under 40 years to almost 40% at ages above 80 years, Fig. 5. A partial solution to improving comparability could be the age-standardised mortality rate, that is the mortality that a population would have if it had the age distribution of a reference population (e.g., the WHO World Standard Population)^47,48^, although it is not without criticism^49^. In this series, the age-standardised mortality rate with this reference population is 2.9 deaths per 100 COVID-19 patients admitted.

*Mortality rate and model performance can be biased in studies with incomplete follow-up,* and cannot be controlled in the analysis phase. In mortality studies published in the first months of the pandemic, it has been frequent to limit the follow-up to 2 weeks of hospital stay; so that only those patients who have died or been discharged during that time were analysed and those who remained hospitalised were excluded^16,18,50^. The lack of follow-up information on these, most likely biases the estimation of crude mortality and the performance of predictive models of mortality^1,33^. Figure 2 shows the distribution of hospital stay in our series depending on the outcome, the group of severe but surviving patients had the longest stay, well above 14 days, so would be largely censored for the analysis if the follow-up be limited to two weeks: as it is a "selective" loss of survivors, it leads to overestimate the mortality rate; and in addition, as it is also a "selective" loss of patients with a difficult prognosis (they were severely ill but survived), it leads to overestimate the performance of the prediction model. Our series only has a 3% loss of included patients, and not related to the length of stay nor outcome, but due to transfer from the Emergency Department to another hospital because of their place of residence.

*We have aimed to keep the predictive model as applicable and straightforward as possible without compromising performance*; that is why we have not included in the final models some laboratory variables that could be in, from a statistical point of view. With the same perspective, our models are advantageous compared with others that require tools with little availability today, such as artificial intelligence or computer applications with copyright^34,36,37,46,47^. This does not mean that all these issues could not be necessary for other objectives and contexts; v.g., comorbidity variables probably be more decisive in countries with an older population; while variables of acute inflammatory damage do so in countries with a young population^29,51,52^.

*What use can these models have?* An essential requirement to apply them with confidence is their validation in independent but representative samples. Once validated, it can have multiple applications, both in the clinical and management area:

- Support to make clinical decisions when, after a routine initial assessment, the course of action is unclear. In many situations it is necessary to filter patients according to severity, for example, to keep as an outpatient or where to ubicate in a hospital. Usually, the most convenient approach is to start with a screening tool to identify those at high risk to get the most from scarce resources. Screening tools are characterized by high sensitivity, and that is the main feature of our model: the global sensitivity for predicting severe disease is 93%, and specificity 55%. The corresponding nomogram in the Supplementary material would allow to calculate the risk for a particular patient, and together with the clinical judgment get to a conclusion. Those patients considered at high risk could be managed with a short observation period to check the tendency and carry out more specific tests that are often more complex and resource-consuming.
- Support in decision-making for the management of the infrastructure necessary for the assistance to function as efficiently and effectively as possible.
- Quality control, through the relationship between observed and expected mortality according to the model^53^.

### Study limitations

The sample came from a single centre during the first months of the pandemic when the standard treatment was not the same as nowadays. Its size is modest, and though sufficient for the study's objective, it still produces wide confidence intervals in relevant variables. There is a potential risk of overfitting in the development of all predictive models, so it is necessary to validate it in an independent sample of patients before any clinical use.

## Conclusion

In patients with COVID-19 pneumonia, the prognosis can likely be significantly narrowed by combining a comorbidity scale and a current severity scale of pneumonia, both based in clinical data readily available. This study proposes a predictive model based on Age-Adjusted Charlson index, the CRB scale, and baseline arterial saturation. It can be completed at the first medical contact through standard anamnesis, physical examination, and a pocket pulse oximeter.

## Supplementary Information

Supplementary Information.

## Data Availability

The datasets supporting the conclusions of this article are included within the article and its additional file. Further details are available from the corresponding author, PNO, upon reasonable request.
