# Supplementary material for: Prognosis of COVID-19 pneumonia can be early predicted combining Age-adjusted Charlson Comorbidity Index, CRB score and baseline oxygen saturation
Source: Sci Rep. 2022 Feb 11;12:2367. doi: 10.1038/s41598-022-06199-3 (PMC8837655; doi:10.1038/s41598-022-06199-3)
Supplement: Supplementary file 1 — Supplementary Information. [file 41598_2022_6199_MOESM1_ESM.docx]

**SUPPLEMENTARY MATERIAL OF MANUSCRIPT: PROGNOSIS OF COVID-19 PNEUMONIA CAN BE EARLY PREDICTED COMBINING AGE-CHARLSON COMORBIDITY INDEX, CRB SCORE AND BASELINE OXYGEN SATURATION.**

**AUTHORS:**

Nuevo-Ortega, Pilar; Reina-Artacho, Carmen; Dominguez-Moreno, Francisco; Becerra-Muñoz, Victor Manuel; Ruiz-Del-Fresno, Luis; Estecha-Foncea, Maria Antonia.

**OBJECTIVE:**

To develop a model to predict which patients with COVID-19 pneumonia are at high risk of developing severe illness, using readily available clinical data in the absence of laboratory or sophisticated computing/artificial intelligence.

**FIGURE S1. DAILY FLOW OF PATIENTS: ADMISSIONS, DISCHARGES, HOSPITALISED. FIRST WAVE COVID-19 PANDEMIC. HUVV.**

**ADDITIONAL INFORMATION ABOUT THE COMPONENTS OF THE MODELS**

The final predictive model for the primary outcome (progression to severe disease) is composed of CRB score,[*[1]*](https://paperpile.com/c/EMWmZx/yLQae) Age-Adjusted Charlson Comorbidity index[*[2]*](https://paperpile.com/c/EMWmZx/Lc53Q) and baseline arterial oxygen saturation by pulse oximetry (SpO_2_); they are scored as shown in the following tables:

*CRB score*

| Variables | Puntos | Comentarios |
| --- | --- | --- |
| Mental confusion | 1 |  |
| Tachypnea > 30 rpm | 1 |  |
| Arterial hypotension | 1 | Systolic < 90 mmHg or diastolic =< 60 mmHg |

*Age-Adjusted Charlson index*

| Variable | Points | Comments |
| --- | --- | --- |
| Myocardial infarction | 1 | One or more previous AMI |
| Congestive heart failure | 1 |  |
| Peripheral vascular disease | 1 | Including aortic aneurysm (diameter > 6 cm) |
| Cerebrovascular disease or transient ischemic attack | 1 | With minor or no sequelae |
| Dementia | 1 | - |
| Chronic obstructive pulmonary disease | 1 | - |
| Connective tissue disease | 1 | - |
| Peptic ulcer disease | 1 | - |
| Mild liver disease | 1 | Chronic hepatitis or cirrhosis without portal hypertension |
| Uncomplicated diabetes | 1 | - |
| Hemiplegia | 2 | Hemiplegia or paraplegia from any cause, including cerebrovascular disease |
| Moderate to severe chronic kidney disease | 2 | Severe: with dialysis, transplanted or uremia.  Moderate: creatinine > 0.27 mmol/L. |
| Diabetes with end-organ damage | 2 | - |
| Localized solid tumor | 2 | - |
| Leukemia | 2 | including chronic and polycythemia vera |
| Lymphoma | 2 | including mieloma |
| Moderate to severe liver disease | 3 | Cirrhosis with portal hypertension |
| Metastatic solid tumor | 6 | - |
| AIDS | 6 |  |
| Age | 1 point for every decade starting at 50 years, until >= 80: | |

*Baseline SpO_2_*

| Variable | Points | Comments |
| --- | --- | --- |
| Baseline SpO_2_ < 90% | 1 | 0 points if it is above 90% |

**Quantifying the individual contribution of each predictor variable to the overall performance of the model.** It is a complex issue since, in general, the information conveyed by the predictor variables is not unique and exclusive to each one. Usually, there is a certain degree of overlap, confusion, interaction, etc., which makes the performance of the global model not the sum of the returns of the individual predictors (it can be lower or higher). In any case, if we accept that Nagelkerke's R squared is an estimator of the overall performance of the model, its value for the models composed of each single predictor or pair of predictors could serve as an approximation (Nagelkerke's R square takes values from 0 to 1, the higher, the better).

| Models | | Nagelkerke’s R square |
| --- | --- | --- |
| One predictor | Age adjusted Charlson Comorbidity index (AgeCharlson) | 0.215 |
|  | CRB | 0.204 |
|  | Baseline SpO_2_ < 90% | 0.272 |
| Two predictors | AgeCharlson & CRB | 0.333 |
|  | AgeCharlson & SpO_2_<90 | 0.359 |
|  | CRB & SpO_2_<90 | 0.356 |
| Three predictors | AgeCharlson & CRB & SpO_2_<90 | 0.421 |

**The general analytical process was the following**. First, we explored age and the rest of the variables as individual predictors in the bivariate analysis (tables 1 to 4 of main manuscript), then carried out intermediate models with up to 5 predictors chosen by clinical meaning to select a parsimonious set of explanatory variables, accepting or discarding them in base to previous clinical knowledge of severely ill patients, and statistical reasons. After deciding the core structure of the model, we did the final multivariate analysis, including just the three essential predictors. Finally, we explored the utility of rescuing some previously discarded variables, but none significantly improved the clinical model, so it remained as shown in tables 5 and 6 of main manuscript.

**Age as a predictor of progression to severe disease in community-acquired pneumonia**. Age has previously been used in all its possible formats: as an individual predictor (continuous, [3] categorized by decades [4] or dichotomized [5]), or integrated into the age-adjusted Charlson comorbidity index as a composite variable.[6] We decided to use it integrated to decrease the number of predictors in the final model (improves precision). And to choose Charlson adjusted for age instead of CRB-65 for two reasons: firstly, because of the affinity of the information transmitted, age is a marker of comorbidity like Charlson (not of acute derangement like CRB), that type of comorbidity that many times is hidden or challenging to measure; and secondly, age-adjusted Charlson takes better advantage of the information contained in age dividing it into five categories rather than two like CRB-65.

**Other variables tested and not included in the main model.** After selecting the main model for the primary objective (progression to severe disease) , composed of three predictors (Age-adjusted Charlson Comorbidity index, CRB score and baseline SpO_2_ on admission), we tested whether some of the previously discarded variables could be relevant predictors added to the main model; in the following table we comment them:

| Gender | Didn´t improved the model. It didn’t reach statistical significance (0.067). No missing data. |
| --- | --- |
| Hypertension | Didn´t improved the model. It didn’t reach statistical significance (0.32). No missing data. |
| Lymphocytes count | Didn´t improved the model. It didn’t reach statistical significance (0.068). 382/392 valid data. |
| D-dimers | Didn´t improved the model. It didn’t reach statistical significance (0.067). 331/392 valid data. |
| C-reactive protein | Didn´t clinically improved the model (Nagelkerke’s square R: 0.430 vs 0.421). It reached statistical significance (0.021). 382/392 valid data. |
| Procalcitonin | Didn´t improved the model (Nagelkerke’s square R: 0.408 vs 0.421). It reached statistical significance (0.027). 264/392 valid data. |
| Lactate dehydrogenase | Didn´t improved the model (Nagelkerke’s square R: 0.421 vs 0.421). It reached statistical significance (0.005). 371/392 valid data. |
| AST | Didn´t improved the model. It didn’t reach statistical significance (0.230). 339/392 valid data. |
| Ferritin | Didn´t improved the model. It didn’t reach statistical significance and distorted the model (0.371). Many missing data on admission (253/392 missing data). |
| Troponin | It reached statistical significance (0.030), didn’t improve the model but distorted it. Many missing data on admission (314/392 missing data). |
| Sodium | Didn´t improved the model. It didn’t reach statistical significance (0.109). 378/392 valid data. |
| Leucocytes count | Didn´t improved the model. It didn’t reach statistical significance (0.997). 382/392 valid data. |
| Hemoglobin | Didn´t improved the model. It didn’t reach statistical significance (0.121). 381/392 valid data. |
| Platelets count | Didn´t improved the model. It didn’t reach statistical significance (0.102). 381/392 valid data. |
| Acute Kidney failure | Didn´t improved the model. It didn’t reach statistical significance (0.859). 380/392 valid data. |
| Delay from symptoms onset to hospital admission | Didn´t improved the model. It didn’t reach statistical significance (0.095). No missing data. |

As a result of this process and to offer all potentially useful information we communicate two secondary models adding laboratory variables, one for the primary and one for the secondary outcome:

Table S1. Clinical-analytical multivariate model for the prediction of severe disease (primary outcome); with the same variables of the Clinical Model (Age-Charlson, CRB and SPO2) plus the two laboratory variables that are statistically significant: high CRP and LDH.

|  |  |  |  | 95%CI for OR | |
| --- | --- | --- | --- | --- | --- |
|  | B | Sig | OR | Lower | Upper |
| Age-Charlson | 0.252 | <0.001 | 1.287 | 1.153 | 1.436 |
| CRB | 1.192 | <0.001 | 3.295 | 1.710 | 6.350 |
| Baseline SpO2 < 90% | 1.636 | <0.001 | 5.132 | 2.512 | 10.487 |
| CRP | 1.527 | 0.053 | 4.603 | 0.977 | 21.677 |
| LDH | 0.748 | 0.029 | 2.113 | 1.080 | 4.134 |
| Constant | -4.549 | <0.001 | 0.011 |  |  |

Nagelkerke’s R square 0.436

Figure S2: ROC Curve of the clinical-analytical predictive model of severe disease.

Table S2. Clinical-analytical multivariate model for prediction of death (secondary outcome), with the same variables of the Clinical Model (Age-Charlson, CRB and SPO2) plus the two laboratory variables that are also statistically significant: high LDH and lymphocyte count.

|  | B | Sig. | OR | 95%CI OR | |
| --- | --- | --- | --- | --- | --- |
|  |  |  |  | Lower | Upper |
| Age-Charlson | 0.405 | <0.001 | 1.500 | 1.285 | 1.751 |
| CRB | 1.094 | 0.002 | 2.988 | 1.498 | 5.959 |
| Baseline SpO2 < 90% | 1.486 | <0,001 | 4.418 | 1.991 | 9.802 |
| Lymphocytes (x10^2^/mL) | -0.079 | 0.036 | 0.924 | 0.858 | 0.995 |
| LDH | 1.117 | 0.028 | 3.055 | 1.130 | 8.261 |
| Constant | -4.420 | <0.001 | 0.012 |  |  |

Nagelkerke’s R square 0.497

Figure S3. ROC curve. Clinical-Analytical model for mortality prediction

**HOW TO CALCULATE THE PROBABILITY OF AN OUTCOME FOR A SPECIFIC PATIENT.**

1. **Numerically**

For any specific patient, the expected probability of developing the outcome can be calculated from the equation of the multivariate model. In the model equation

$$Ln\left( \frac{p}{1-p} \right)=Constant+\beta_{AgeCharlson}\times AgeCharlson+\beta_{CRB}\times CRB+\beta_{{SpO}_{2}}\times{SpO}_{2}$$

First substitute "AgeCharlson ", "CRB" and "SpO2" by the values of the patient, and Constant and 𝛃 coefficients by their respective values from the tables of the model (Table 5 of severe disease, or Table 6 for death), then clearing the p.

*Example 1.* A patient of 48 years without comorbidity, that getting to the emergency room is oriented without tachypnea nor hypotension, and has a SpO2 of 95%, has 0 points in all predictors (Age-Charlson, CRB and SpO2), the expected probability of developing severe illness or death is:

$$p_{Severe Disease} =\frac{e^{-2.676 + 0.253\times0 + 1.427\times0 + 1.866\times0}}{1 +e^{-2.676 + 0.253\times0 + 1.427\times0 + 1.866\times0}}=6.4\%$$

$$p_{Death} =\frac{e^{-4.609 + 0.424\times0 + 1.091\times0 + 1.636\times0}}{1 +e^{-4.609 + 0.424\times0 + 1.091\times0 + 1.636\times0}}=0.9\%$$

*Example 2.* The average patient in the series has 2 points in Age-Charlson, and 0 in CRB scale and SpO2, so the expected probability of severe illness is 10%, and of dying 2%.

$$p_{Severe Disease} =\frac{e^{-2,676 + 0,253\times2 + 1,427\times0 + 1,866\times0}}{1 +e^{-2,676 + 0,253\times2 + 1,427\times0 + 1,866\times0}}=10,2\%$$

$$p_{Death} =\frac{e^{-4,609 + 0,424\times2 + 1,091\times0 + 1,636\times0}}{1 +e^{-4,609 + 0,424\times2 + 1,091\times0 + 1,636\times0}}=2,2\%$$

1. **Graphically, with nomograms**

With the following nomograms, we could calculate a particular patient's probability for developing severe disease or eventually die. Firstly, translate the punctuation of the two scores and pulse oximetry to risk points in the upper axis (labelled ‘Points’), then sum them up (axis labelled 'Total Points'), and finally, look for the risk corresponding to that total in the lower axis ('Risk of …).

- 1. Nomogram to calculate the risk of developing disease

- 1. Nomogram to calculate the risk of death

**TRIPOD CHECKLIST: PREDICTION MODEL DEVELOPMENT.** **[7]**

| **Section/Topic**  **It** | | **Checklist Item** | **Page** |
| --- | --- | --- | --- |
| **Title and abstract** | | | |
| Title | 1 | Identify the study as developing and/or validating a multivariable prediction model, the target population, and the outcome to be predicted. | 1 |
| Abstract | 2 | Provide a summary of objectives, study design, setting, participants, sample size, predictors, outcome, statistical analysis, results, and conclusions. | 2 |
| **Introduction** | | | |
| Background and objectives | 3a | Explain the medical context (including whether diagnostic or prognostic) and rationale for developing or validating the multivariable prediction model, including references to existing models. | 3 |
|  | 3b | Specify the objectives, including whether the study describes the development or validation of the model or both. | 3 |
| **Methods** | | | |
| Source of data | 4a | Describe the study design or source of data (e.g., randomized trial, cohort, or registry data), separately for the development and validation data sets, if applicable. | 4 |
|  | 4b | Specify the key study dates, including start of accrual; end of accrual; and, if applicable, end of follow-up. | 4 |
| Participants | 5a | Specify key elements of the study setting (e.g., primary care, secondary care, general population) including number and location of centres. | 4 |
|  | 5b | Describe eligibility criteria for participants. | 4 |
|  | 5c | Give details of treatments received, if relevant. | NA |
| Outcome | 6a | Clearly define the outcome predicted by the model, how and when assessed. | 5 |
|  | 6b | Report any actions to blind assessment of the outcome to be predicted. | - |
| Predictors | 7a | Clearly define all predictors used in developing or validating the multivariable prediction model, including how and when they were measured. | 5-6 |
|  | 7b | Report any actions to blind assessment of predictors for the outcome and other predictors. | - |
| Sample size | 8 | Explain how the study size was arrived at. | 6 |
| Missing data | 9 | Describe how missing data were handled (e.g., complete-case analysis, single imputation, multiple imputation) with details of any imputation method. | 6 |
| Statistical analysis methods | 10a | Describe how predictors were handled in the analyses. | 6 & Supp |
|  | 10b | Specify type of model, all model-building procedures (including any predictor selection), and method for internal validation. | 6 & Supp |
|  | 10d | Specify all measures used to assess model performance and, if relevant, to compare multiple models. | 6 & Supp |
| Risk groups | 11 | Provide details on how risk groups were created, if done. | NA |
| **Results** | | | |
| Participants | 13a | Describe the flow of participants through the study, including the number of participants with and without the outcome and, if applicable, a summary of the follow-up time. A diagram may be helpful. | 7 |
|  | 13b | Describe the characteristics of the participants (basic demographics, clinical features, available predictors), including the number of participants with missing data for predictors and outcome. | 8 - 14 |
| Model development | 14a | Specify the number of participants and outcome events in each analysis. | 8 - 14 |
|  | 14b | If done, report the unadjusted association between each candidate predictor and outcome. | 8 - 14 |
| Model specification | 15a | Present the full prediction model to allow predictions for individuals (i.e., all regression coefficients, and model intercept or baseline survival at a given time point). | 15-16 |
|  | 15b | Explain how to the use the prediction model. | Supp |
| Model performance | 16 | Report performance measures (with CIs) for the prediction model. | 15-16 |
| **Discussion** | | | |
| Limitations | 18 | Discuss any limitations of the study (such as nonrepresentative sample, few events per predictor, missing data). | 22 |
| Interpretation | 19b | Give an overall interpretation of the results, considering objectives, limitations, and results from similar studies, and other relevant evidence. | 17-23 |
| Implications | 20 | Discuss the potential clinical use of the model and implications for future research. | 22-23 |
| **Other information** | | | |
| Supplementary information | 21 | Provide information about the availability of supplementary resources, such as study protocol, Web calculator, and data sets. | Supp |
| Funding | 22 | Give the source of funding and the role of the funders for the present study. | NA |

In the “Item” column, in green background those items successfully completed, in yellow those non accomplished, and in white those non-applicable.

**COLLABORATORS:**

- Murcia-Casas, B. Collected data and provided and cared for study patients.
- Martinez-Mesa, A. Collected data and provided and cared for study patients.
- Cabrera-Cesar, E. Collected data and provided and cared for study patients.
- Gomez-Perez, AM. Collected data and provided and cared for study patients.
- Aguilar-Galvez, AM. Collected data and provided and cared for study patients.
- Doncel-Abad, V. Collected data and provided and cared for study patients.
- Rodriguez-Capitan, J. Collected data and provided and cared for study patients.
- Vera-Sanchez, MC. Collected data and provided and cared for study patients.
- Gonzalez-Redondo, P. Collected data and provided and cared for study patients.
- Sanchez-Alvarez, E. Collected data and provided and cared for study patients.
- Puerto-Morlan, A. Provided and cared for study patients.
- Zamboschi, NA. Provided and cared for study patients.
- De La Torre-Muñoz, A. Provided and cared for study patients.
- Nieto-Gonzalez, M. Provided and cared for study patients.
- Moratalla-Cecilia, G. Provided and cared for study patients.
- Segura-Gonzalez, F. Provided and cared for study patients.
- Martinez-Lopez, P. Provided and cared for study patients.
- Salido-Diaz, L. Provided and cared for study patients.
- Cordon-Alvarez, S. Provided and cared for study patients.
- Daga-Ruiz, D. Provided and cared for study patients.
- Salazar-Ramirez, C. Provided and cared for study patients.
- Rueda-Molina, C. Provided and cared for study patients.
- Mateos-Rodriguez, M. Provided and cared for study patients.
- Sanchez-Calderon, A. Provided and cared for study patients.
- Sanchez-Garcia, A. Provided and cared for study patients.
- Fernandez-Villalba, A. Provided and cared for study patients.
- Garcia-Gomez, IG. Provided and cared for study patients.
- Cota-Delgado, C. Provided and cared for study patients.
- Vallejo-Baez, A.Provided and cared for study patients.
- Castillo-Caballero, JM. Provided and cared for study patients.
- Valera-Rubio, M. Provided and cared for study patients.
- Lara-Dominguez, P. Provided and cared for study patients.
- Estevez-Escobar, E. Provided and cared for study patients.
- Garcia-Aragon, S. Provided and cared for study patients.
- Perez-Lopez, C. Provided and cared for study patients.
- Barrera-Serrano, R. Provided and cared for study patients.
- Guerrero-Orriach, JL Provided and cared for study patients.

**REFERENCES**

1. [Bauer TT, Ewig S, Marre R, Suttorp N. CRB‐65 predicts death from community‐acquired pneumonia. J Intern Med. 2006. Available:](http://paperpile.com/b/EMWmZx/yLQae) <https://onlinelibrary.wiley.com/doi/abs/10.1111/j.1365-2796.2006.01657.x>

2. [Charlson M, Szatrowski TP, Peterson J, Gold J. Validation of a combined comorbidity index. J Clin Epidemiol. 1994;47: 1245–1251. doi:](http://paperpile.com/b/EMWmZx/Lc53Q)[10.1016/0895-4356(94)90129-5](http://dx.doi.org/10.1016/0895-4356(94)90129-5)

3. Fine MJ, Auble TE, Yealy DM, Hanusa BH, Weissfeld LA, Singer DE, et al. A prediction rule to identify low-risk patients with community-acquired pneumonia. N Engl J Med. 1997;336: 243–250.

4. Charlson M, Szatrowski TP, Peterson J, Gold J. Validation of a combined comorbidity index. J Clin Epidemiol. 1994;47: 1245–1251.

5. Ewig S, Birkner N, Strauss R, Schaefer E, Pauletzki J, Bischoff H, et al. New perspectives on community-acquired pneumonia in 388 406 patients. Results from a nationwide mandatory performance measurement programme in healthcare quality. Thorax. 2009;64: 1062–1069.

6. Kim DH, Park HC, Cho A, Kim J, Yun K-S, Kim J, et al. Age-adjusted Charlson comorbidity index score is the best predictor for severe clinical outcome in the hospitalized patients with COVID-19 infection. Medicine . 2021;100: e25900.

7. [Collins GS, Reitsma JB, Altman DG, Moons KGM. Transparent Reporting of a multivariable prediction model for Individual Prognosis Or Diagnosis (TRIPOD): the TRIPOD Statement. Br J Surg. 2015;102: 148–158. doi:](http://paperpile.com/b/EMWmZx/R4YO)[10.1002/bjs.9736](http://dx.doi.org/10.1002/bjs.9736)
